# The Assessment of Organophosphate Pesticide Exposure among School Children in Four Regions of Thailand: Analysis of Dialkyl Phosphate Metabolites in Students’ Urine and Organophosphate Pesticide Residues in Vegetables for School Lunch

**DOI:** 10.3390/toxics10080434

**Published:** 2022-07-29

**Authors:** Anurak Wongta, Nootchakarn Sawang, Phanika Tongjai, Marut Jatiket, Surat Hongsibsong

**Affiliations:** 1 Environmental, Occupational and NCD Center of Excellent, Research Institute for Health Sciences, Chiang Mai University, Chiang Mai 50200, Thailand; anurakwongta@gmail.com (A.W.); nootchakarn_s@cmu.ac.th (N.S.); phannika_t@hotmail.com (P.T.); 2 Department of Community Medicine, Faculty of Medicine, Chiang Mai University, Chiang Mai 50200, Thailand; 3 School of Health Science Research, Research Institute for Health Sciences, Chiang Mai University, Chiang Mai 50200, Thailand; 4 Thai Education Foundation, Lampoon 51150, Thailand; marutj@thaied.org

**Keywords:** organophosphate, dialkyl phosphates metabolites, school children, pesticides exposure, pesticides residues

## Abstract

In Thailand, pesticides containing organophosphates (OP) are frequently applied to crops to suppress insects. School children can be exposed to OPs on a daily basis, from food consumption to breathing and touching pesticides drifted near classrooms. Living in an agricultural area can also be one of the causes. As a result, it is important to monitor OPs residues in the food chain and biomarkers of exposure. The Gas Chromatography–Flame Photometric Detector method was employed to examine the relationship between OPs residue and DAPs (Diakly phosphate) in four targeted locations in Thailand, as well as to examine the residues of OPs in vegetable samples and DAPs in 395 school children’s urine samples. Vegetables were found to contain at least one OP, with chlorpyrifos being the most prevalent. The OPs detected frequencies for Sakon Nakhon, Chiang Mai, Phang Nga, and Pathum Thani are 96.1%, 94%, 91.7%, and 83.3%, respectively. The overall centration level of OPs showed 0.3261 mg/kg, 0.0636 mg/kg, 0.0023 mg/kg, 0.0150 mg/kg, 0.2003 mg/kg, 0.0295 mg/kg, and 0.0034 mg/kg for diazinon, dimethoate, pirimiphos-methyl, chlorpyrifos, profenofos, ethion, and triazophos, respectively. Nearly 98% of school children were detected with at least one DAP. The overall level of dimethyl phosphate metabolites (5.258 µmole/mole creatinine) in urine samples is higher than diethyl phosphate metabolites (2.884 µmole/mole creatinine), especially in the case of Pathum Thani. Our findings show a consistent relationship between OPs in vegetables from wet markets and DAPs in urine samples of school children in various parts of Thailand.

## 1. Introduction

Thai farmers frequently employ organophosphate (OP) pesticides to manage insects on their crops. Children exposed to pesticides are more likely to have cancer, respiratory issues, reproductive, endocrine, skin, and neurological disorders [1,2,3,4,5,6]. Since most of their organs are still developing, school children are more susceptible to pesticide exposure than adults are, as pesticides can affect the growth of different organs. They are also more vulnerable because of their distinct daily activity patterns and physiological traits [7,8,9]. They are more sensitive to the potential neurotoxic effects of pesticides because of their organ systems, such as the brain and central nervous system. These organs develop rapidly in those years of growth; therefore, they lack levels of pesticides that detoxify enzymes. It is well understood that acute effects of exposure to the OPs produce a wide range of neurological symptoms, and it can be monitored by clinical signs and inhibition of acetylcholinesterase activity [10]. Moderate or low exposure to pesticides is suspected of having adverse health effects as previous studies have reported that prolonged exposure to OPs can cause oxidative stress and damage DNA strands, resulting in an increased risk for chronic diseases such as cancer and neurological diseases [11].

School children can be exposed to various OPs daily, from daily intake, breathing, touching pesticides drift near classrooms, engaging in farm activity, or living nearby agricultural areas being sprayed. Common routes of exposure include contact with skin, inhalation, and dietary intake [12,13]. In addition, children’s health risks were affected by environmental injustice, especially among poor, minority, and marginalized populations for several reasons: toxic chemicals, contaminated air, contaminated water, unsafe workplaces, and other environmental hazards [14]. Prior research indicates that children are particularly susceptible to the potentially harmful health effects of pesticides are irreversible and may cause public health concerns. The public and policymakers must therefore have access to information on sources and exposure characteristics in order to lower exposure risks for children and safeguard their health. The government has allocated money for school lunches for students attending government schools, particularly in Thailand. The school is in charge of overseeing the administration of the kitchen and purchasing ingredients, primarily from nearby wet markets, including produce, fruit, and meat. Despite this, OP residues have been identified in fruit and vegetable samples from wet markets in various investigations [15,16,17]. Due to the fact that each DAP can be produced from multiple OP pesticides, dialkyl phosphates (DAPs) are metabolites that can be employed as non-specific urine biomarkers of OP pesticides. It was widely used for assessing exposure to OPs from several routes, that is, oral, dermal, and inhalation. Once OP pesticide exposure occurs, it is metabolized via dealkylation, hydrolysis, and isomerization. DAPs in the urine can be used indirectly to measure OP pesticide exposure [18,19]. In addition, dimethyl OP pesticides produce only dimethyl phosphate (DM) metabolites, while diethyl OP pesticides produce diethyl phosphate (DE) metabolites [20]. Therefore, most studies grouped the metabolites into these two groups (DM and DE) and indicated urine DAP concentrations as biomarkers of exposure to OPs within hours or days after sampling [21]. Studies reported the exposure in children using DAPs as the biomarkers of OPs and showed various risk factors of exposure. In the study by Bradman et al., they reported that children living in the agricultural area were more likely to expose to OPs from multiple sources, and urinary DAPs, particularly DMs levels increased with age, food, and pesticide used region [22]. Moreover, children from agricultural families in the northern part of Thailand were observed, and dietary sources were the likely contributors to pesticide exposure [23].

School children have the risk of exposure to pesticides for the reasons mentioned above, especially organophosphates, which are the most commonly used pesticides in Thailand. Therefore, the aim of this study was to investigate the relationship between the levels of OPs metabolites in urine samples of school children by using DAPs as the biomarker and the levels of OPs residues in vegetables from the market where the school is located in four target areas.

## 2. Materials and Methods

### 2.1. Study Design

A cross-sectional study was conducted on school children 5–15-year-old from 4 provinces in 4 parts of Thailand, i.e., Chiang Mai (northern), Sakon Nakhon (north-eastern), Pathum Thani (middle), and Phang Nga (southern) province. Demographic data were gathered. For age, data were collected through interviews, and for weight and height, data were collected through weight and height measurements.

The research project was approved by Human Experimentation Committee (HEC), Research Institute for Health Sciences, Chiang Mai University (Certificate no. 8/60, 18 October 2017).

### 2.2. Sample Collection

The OPs residues in vegetables were used as the contaminating markers, and DAP metabolites in urine were used as biomarkers of exposure among school children:

(1) Vegetable samples: Two hundred and twenty-two vegetable samples were purchased from the same local wet markets where the cooker usually buys their ingredients, i.e., Chinese kale (*Brassica alboglabra L.H. Bailey*), cabbage (*Brassica oleracea var. capitata* L.), Chinese cabbage (*Brassica rapa* L. (*Brassica pekinensis var. cylindrica Tsen and S.H.Lee*)), Mockpak choi (*Brassica rapa* L.), carrot (*Daucus carota*), water convolvulus (*Ipomoea aquatica Forsk. Var. reptan*) yellow berried nightshade (*Solanum xanthocarpum Schrad. and Wendl*.), tomato (*Lycopersicon esculentum Mill*.), and yard long bean (*Vigna unguiculata ssp. Sesquipedalis*);
(2) Urine samples: Three hundred ninety-five first-morning void urine samples were collected in the period of 5–8 AM from the children’s house and kept in polyethylene bottles with a unique identifying code of an individual. Furthermore, the bottles were secured in zip-lock plastic bags and kept in an ice box while transporting them for testing.

The vegetable and urine samples were transferred to the laboratory of Environmental and Occupational Health Sciences, Research Institute for Health Sciences (RIHES), Chiang Mai University, Chiang Mai, Thailand. The vegetable samples were finely chopped and stored at −20 °C in a freezer using a zip-lock plastic bag with individual code labeling. The urine samples were aliquoted in three tubes (10 mL in 15 mL-cryo-tube) for DAPs and creatinine analysis, then stored at −20 °C in a freezer prior to analysis.

### 2.3. Organophosphate Pesticide Analysis of Vegetables

Sample extraction and analysis were modified from the method of Koesukwiwat et al. [24] and Sapbamrer and Hongsibsong [15]. Briefly, 5 g of vegetable samples were added into a 50 mL centrifuge tube containing 10 mL acetonitrile (high-performance liquid chromatography [HPLC] grade; JT Baker, USA). The volume of 250 μL of 5 μg/mL triphenylphosphate (internal standard [IS]) was added, and centrifuging was performed at 2500 rpm for 5 min. The supernatant was transferred to a new 50 mL centrifuge tube that contained 6 g of MgSO_4_ and 3 g of NaCl and was subsequently centrifuged at 2500 rpm for 5 min. The evaporation was performed to complete the dryness of the extracted solution. A vacuum rotary evaporator (Buchi, Switzerland) with a water bath at 30 to 35 °C was used. The pellet was then resuspended with 5 mL of ethyl acetate (HPLC grade; JT Baker, Phillipsburg, NJ, USA). The volume of 1 mL of ethyl acetate phase was pipetted to a dispersive solid-phase extraction tube (carbon black, Vertical Chromatography Co., LTD, Nonthaburi, Thailand) and centrifuged at 2000 rpm for 3 min. The extract was evaporated with a gentle stream of nitrogen at room temperature and resuspended by 0.5 mL of ethyl acetate for gas chromatography (GC) analysis. The GC analysis consisted of a Hewlett-Packard model 6890 equipped with a flame photometric detector, a capillary column (DB-5 MS, 0.25 mm × I.D. × 30 m length × 0.25 μm film thickness (Agilent J and W column; Agilent Technologies, Santa Clara, CA, USA)), and a computerized data handling system (GC Chemstation A.10.02; Agilent Technologies, Santa Clara, CA, USA). The injection port had a temperature of 220 °C (splitless mode). The oven temperature was programmed as follows: initial temperature of 100 °C for 10 min, the first ramp of 15 °C/min to 180 °C (5 min), the second ramp of 5 °C/min to 250 °C (3 min), and final temperature of 290 °C for 4 min. The carrier gas was 99.999 % helium.

The method’s precision, accuracy, and overall reliability were assessed using quality control data for 7 types of OPs including diazinon (Dr. Ehrenstorfer GmbH, Augsburg, Germany), dimethoate (Dr. Ehrenstorfer GmbH, Augsburg, Germany), pirimiphos-methyl (Dr. Ehrenstorfer GmbH, Augsburg, Germany), chlorpyrifos (Dr. Ehrenstorfer GmbH, Augsburg, Germany), profenofos (Dr. Ehrenstorfer GmbH, Augsburg, Germany), ethion (Dr. Ehrenstorfer GmbH, Augsburg, Germany), and triazophos (Dr. Ehrenstorfer GmbH, Augsburg, Germany). Each set of three vegetable samples was subjected to spike the sample recovery for low (0.020 mg/kg), medium (0.080 mg/kg), and high (0.320 mg/kg) concentrations of OPs standard and blank analysis. The mean recovery of spiked Ops standard ranges from 91.31% to 106.53%, 84.45% to 95.85%, and 91.25% to 100.55% for low, medium, and high spiked concentration, respectively. The within-series imprecision (CV%) ranges from 6.05% to 8.6%. The limit of detection (LOD) and quantitative limit (LOQ) were 0.001 mg/kg and 0.004 mg/kg for dimethoate, 0.002 mg/kg and 0.009 mg/kg for diazinon, 0.007 mg/kg and 0.003 mg/kg for pirimiphos-methyl, 0.001 mg/kg and 0.002 for chlorpyrifos, 0.001 mg/kg and 0.003 mg/kg for profenofos, 0.0002 mg/kg and 0.001 mg/kg for ethion, 0.001 mg/kg and 0.005 mg/kg for triazophos

### 2.4. Urinary Dialkylphosphate Metabolites Analysis

A urine sample of 395 students from 4 provinces was randomized. The research was conducted by the Institute of Health Sciences Research. Chiang Mai Research for OP pesticide in 6 types of biomarkers, namely DMP, DMTP, DMDTP, DEP, DETP, and DEDTP. Urinary DAPs were measured using the method of Prapamontol et al. [25], and some were modified from Wongta et al. [17]. The DAPs were analyzed using a Hewlett-Packard 7890B-flame photometric detector (GC-FPD) and 7693 Autosampler (Agilent Technology, Santa Clara, CA, USA) (Agilent Technology, Santa Clara, CA, USA) equipped with HP-5 (30 m × 0.25 mm. id, 0.25 um film thickness) columns. The analysis included six nonspecific metabolites of organophosphates pesticides, that is, DAPs derivatives consisting of dimethyl phosphate (DMP, 98%, TRC canada, Toronto, Canada), diethyl phosphate (DEP, 99.5%, TRC canada, Toronto, Canada), dimethyl thiophosphate (DMTP, >90%, TRC canada, Toronto, Canada), dimethyl dithiophos phate (DMDTP, >90%, TRC canada, Toronto, Canada), diethyl dithiophosphate (DEDTP, 90%, TRC canada, Toronto, Canada), diethyl thiophosphate (DETP, 98%, TRC canada, Toronto, Canada). Sum variables were calculated: sumDes: DEP + DETP + DEDTP, sumDMs: DMP + DMTP + DMDTP, sumDAP: sumDEs + sumDMs. The DAPs concentrations were adjusted for creatinine concentrations and converted from μg/L to μg/g creatinine and μg/L to μmol/mol creatinine. The creatinine in urine was determined by Jaffe’s reaction.

For DAPs, each set of three samples was subjected to spike sample recovery for low (1–25 µg/L), medium (4–75 µg/L), and high (16–125 µg/L) concentrations of OPs standard and urine blank analysis. Pooled urine from a healthy volunteer who had not been given any medicines or directly exposed to chemicals was utilized as a blank for spike recoveries. The mean recovery of spiked DAPs standard ranges from 104.08% to 120.97%, 82.24% to 116.79%, and 80.34% to 102.34% for low, medium, and high spiked concentration, respectively. The within-series imprecision (CV%) ranges from 7.80% to 9.18%. The limit of detection (LOD) and quantitative limit (LOQ) were 2.25 and 20.0 µg/L for DMP, 0.20 and 5.50 µg/L for DMTP, 0.10 µg/L and 3.00 µg/L for DMDTP, 0.05 µg/L and 2.00 µg/L for DEP, 0.10 µg/L and 2.00 µg/L for DETP, 0.10 µg/L and 3.00 µg/L for DEDTP.

### 2.5. Data Analysis

The collected data were analyzed using SPSS Statistics Base 17.0, Thailand. All data were tested for normality before appropriate statistical analyses were performed. Mean, standard deviation (SD), and frequency was reported for variables associated with participant demographics and characteristics. An Independent T-Test was used to assess the difference between the demographics. Chi-square tests (χ^2^) were used for the comparison of categorical data between participant groups. The OPs residues and urinary metabolites were reported as means, SD, median and interquartile range (IQR). The Kruskal–Wallis test was used to compare the urinary OP metabolites in urine samples of school children between the provinces and for OPs residue in vegetable samples between the provinces at the significance level of 0.05.

## 3. Results

The demographic characteristics of the participants are displayed below. There are 395 participants, including 231 males and 164 females, both of whom are the same age (9.73 ± 2.74 and 9.50 ± 2.63, respectively). The BMI, height, and weight all fall within the same range as that displayed in Table 1.

### 3.1. Biomarkers of Organophosphate Pesticide Residues and Exposure

#### Organophosphate Residue in Vegetable Samples

**Figure 1:**
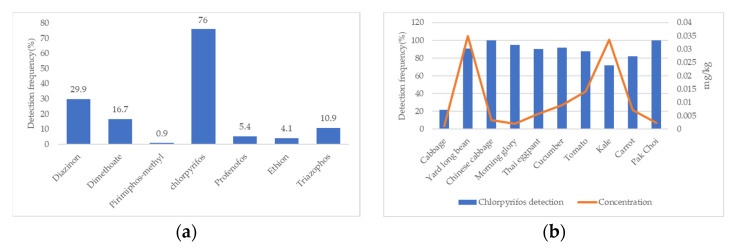

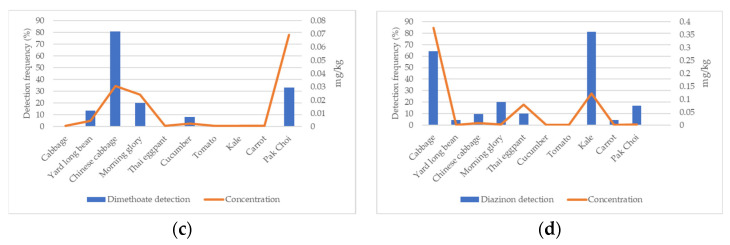
Detection of OPs and concentration of residues in vegetables. Detection frequency (**a**), correlation of detection and concentration of residues; chlorpyrifos (**b**), dimethoate (**c**), and diazinon (**d**).

Two hundred twenty two vegetable samples were tested including cabbage (*n* = 42), yard long bean (*n* = 22), Chinese cabbage (*n* = 31), morning glory (*n* = 20), Thai eggpant (*n* = 20), cucumber (*n* = 12), tomato (*n* = 8), kale (*n* = 32), carrot (*n* = 22), and pak choi (*n* = 12). Chlorpyrifos, diazinon, and dimethoate were shown as the first (76%), second (29.9%), and third (16.7%) detected residues in this study (Figure 1a).

The correlation of detection and concentration of residues based on vegetable type revealed that chlorpyrifos had a high detection percentage in almost all vegetables. (81.8–100%) and showed low concentration except for yard long bean and kale (0.0035 and 0.0034 mg/kg) (Figure 1b). While dimethoate was found in Chinese cabbage (80.6%, 0.0308 mg/kg) (Figure 1c), high detection and concentration for diazinon was also found in just cabbage (64.4%, 0.3761 mg/kg) and kale (81.2%, 0.1229 mg/kg) (Figure 1d).

The province’s review of OPs residue results revealed a similar pattern to the overall detection result (Table 2). Briefly, the most detected is chlorpyrifos in any province, followed by diazinon and dimethoate. However, there is a difference between the kind of detected vegetables and the type of residue in each. The most detected OPs residues were found in Sakon Nakhon (96.1%), followed by Chiang Mai (94%), Phang Nga (91.7), and Pathum Thani (83.3%).

According to the results of the detected vegetable tests, the vegetables with at least one residue were found in Sukhothai to be cabbage, Chinese cabbage, morning glory, and Thai eggplant; in Chiang Mai, it was yard long bean, Chinese cabbage, kale, and carrot; in Phang Nga, it was Chinese cabbage, Thai eggplant, and cucumber; and in Pathum Thani, it was morning glory and pak choi.

According to each province, the frequency of detection and the concentration of OP residues were determined for all vegetables in each province, including 50 samples for CM, 60 samples for PT, 51 samples for SK, and 60 samples for PNG, and the results were displayed in Table 3. The detection showed a similar pattern of vegetable results in each province. Chlorpyrifos was the most detected in all targeted areas at 78%, 61%, 86.3%, and 80% for Chiang Mai (CM), Sakon Nakhon (SK), Pathum Thani (PT), and Phang Nga (PNG) province, respectively. Diazinon was found to be the second in PT and SK (40% and 39.2%), while CM and PNG were triazophos and dimethoate (32% and 21.7%). However, some OPs residues could not be detected in each target area, including pirimiphos-methyl in CM and PT, ethion in PT, profenofos, and triazophos in PNG. The differences in the median of OPs residues between the province were observed; dimethoate was found to be significantly higher in PT (0.1637 mg/kg) than in CM and PNG (0.0114 and 0.0193 mg/kg). In contrast, chlorpyrifos in PT (0.0021 mg/kg) was found significantly lower than in CM, PNG and SK (0.0031, 0.0029 and 0.0025 mg/kg).

### 3.2. Dialkyphosphate Metabolites in Urine Samples

The results showed that about 98% of students detected pesticide residues in the urine (Table 4). DETP (92.8–95.0%) was found highest detected in any province followed by DEP (56.7–80.8%), DMTP (31.1–47.4%), DEDTP (8.2–19.4%), DMDTP (7.1–17.2%), and DMP (3.1–15.8%). The differences in the median of DAPs biomarkers between the province were observed; DMP in PT (16.376 µmole/mole creatinine) was found to be significantly higher than those found in CM (5.053 µmole/mole creatinine), DMDTP in SK (1.182 µmole/mole creatinine) was found higher than CM (0.231 µmole/mole creatinine), while the DEP level in CM (1.635 µmole/mole creatinine) showed higher than in SK (0.735 µmole/mole creatinine). For the sum of DAPs, the results showed just the DAPs level in CM (2.609 µmole/mole creatinine) higher than those in SK (1.596 µmole/mole creatinine).

In order to understand the relation and consequences of OPs contamination and exposure to children in Thailand, the children in government schools were chosen as the school has the same lunch-supported policy. Additionally, almost all the schools buy the food materials from local wet markets, especially vegetables, which were reported of high pesticide residues. DAP metabolites can be produced from several intact forms of OPs, and they can be categorized into DM and DE forms. However, some OP does not produce any DAP metabolites. The detected OPs and DAPs degradation products in the urine are shown in Table 5.

**Figure 2:**
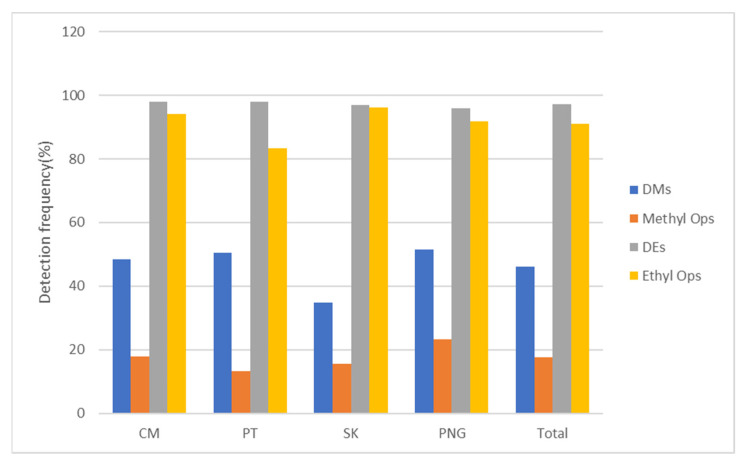
Detection frequency of OP residues and DAP metabolites by province.

**Figure 3:**
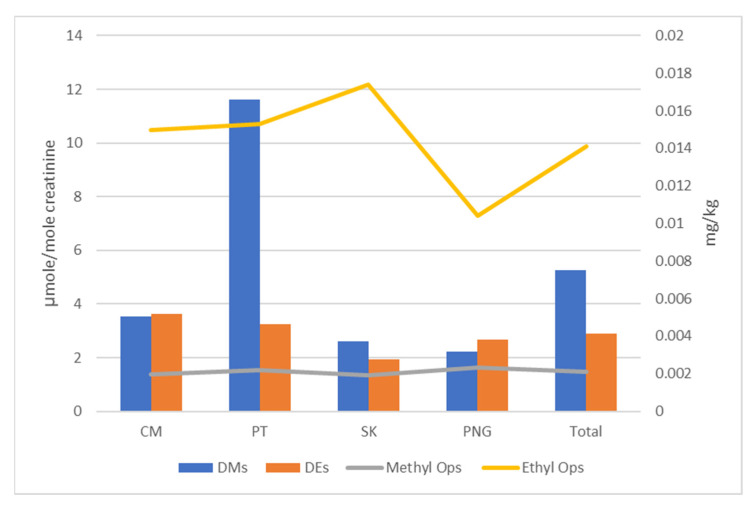
Comparison of DAPs levels in urine samples (bar chart) and OPs residues levels in vegetable samples (line chart) by province.

The relationship between OP residues in vegetables in local wet markets and DAPs in urine samples of school children was observed in this present study. The detection frequency of OP residues and DAPs were constructed in a bar chart to observe the relationship between OP residues and DAPs in each target area, as shown in Figure 2. The result confirmed the high detected DE metabolites consequences from high detected ethyl OP residues and low detected DM metabolites resulting from low detected methyl OP residues. While the overall concentration level of DM metabolites in urine samples is higher than DE metabolites even though the methyl OP residues levels are lower than ethyl OP residues, especially for PT, as shown in Figure 3.

## 4. Discussion

In Thailand, during the past ten years, OPs have been the most widely used pesticides, particularly chlorpyrifos, until it was outlawed in 2020 [22]. However, it appears that today, over all of Thailand, chlorpyrifos and other OP group pesticides are the most often utilized. Several past studies reported OP residues in various types of vegetables, but most of them were studied just in one target area. The report on the finding of OP residues in vegetables from different sources such as farms, markets, and a supermarket in Phayao province, northern Thailand, showed that 59.3% of farms and 13.2% of markets vegetables contained OP residues at or above the maximum residue limits established by the European Union. The most common OP residues detected in farms, markets, and supermarket samples were chlorpyrifos at 50.0%, 33.9%, and 33.3%, respectively [15]. Moreover, in the study by Silipaunyo et al., they reported OP residues were detected at 21.43% in fruit samples and 31.50% in vegetable samples collected from wet markets and supermarkets in Ching Mai province, northern Thailand [27]. The study in Trang province, southern Thailand, reported that 41.58% of 190 samples were contaminated with OPs and carbamates such as cilantro, kale, Chinese cabbage, cabbage, cauliflower, chili, celery, spring onion, yard long bean, cucumber, tomato, Thai eggplant, white radish, and lemon [28].

The present study is the first study that collected vegetable samples from four provinces that represent four regions of Thailand, including Chiang Mai, Sakon Nakhon, Pathum Thani, and Krabi provinces, which represent the northern, north-eastern, middle, and southern parts, respectively. The results of this investigation on OP residues in vegetables show that all samples contained at least one OP residue and that the earlier studies had also revealed chlorpyrifos, diazinon, and dimethoate as the first (76 percent), second (29.9 percent), and third (16.7%) OP residues. The highest concentration of chlorpyrifos found in this study was 0.4936 mg/kg in cabbage from a wet market, which is lower than the levels found in studies by Sapbamrer and Hongsibsong that found 2.423 mg/kg in lemon balm from a farm and 7.785 mg/kg in market-purchased garlic but higher than 0.027 mg/kg in parsley from a grocery store. Most OPs residues were discovered in the province of SK (96.1%), followed by CM (94%), PNG (91.7%), and PT (83.3%). Additionally, 86.3%, 78%, 80%, and 61% for SK, CM, PNG, and PT, respectively, of all target regions were found to have chlorpyrifos. The results did not distinguish across the provinces in terms of total OP residue detection. The quantities of each residue do vary, though, and dimethoate was discovered to be substantially higher in PT than in CM and PNG. As opposed to CM, PNG, and SK, chlorpyrifos levels in PT were found to be substantially lower. That may confirm the explanation that the level of exposure to OP pesticides in consumers depends on the vegetable source, vegetable type, and treatment process [17,29].

The DAPs were used as urinary biomarkers of OP exposure in several previous studies [12,15,17,20,21,22]. In this study, at least one DAP was observed in almost all school children, and there were no differences in the detection between four target areas. However, a difference in concentration levels between the target areas was observed. The overall results found that the DAPs level of school children in SK province (median = 1.596 µmole/mole creatinine) is lower than the others province and significantly lower than those in CM province (median = 2.609 µmole/mole creatinine), especially for DEP metabolite. The DAPs levels in CM province were also reported to be higher levels than in other areas in the previous study [21], and the DAPs levels in SK were also reported to be lower than in children who lived in central Thailand [30,31].

According to a previous study conducted on American residents aged 6–59, DM levels were almost two times greater than DEs levels overall, and they noted that DAP concentrations in children aged 6 to 11 were significantly higher than in adults and adolescents [32]. The province of PT in central Thailand has the largest concentration of DMs. In line with a prior study in the same province that saw school-aged kids residing in a rice and aquaculture farming community [30]. The fact that this location is suburban and close to the capital, as opposed to other areas, where people live in rural settings, may be the source of the higher amount. It would be a good idea to look into this matter more in the future.

Few research discusses the connections between DAPs, particularly in children, and OP residues in local markets. The majority of them investigated contamination, exposure, or the danger of exposure and its repercussions. Several investigations [17,31,32] revealed residues in diverse target areas. In the study by Muoz-Quezada et al., which was conducted in Talca Province, Chile, more than 70% of children consume vegetables from the local market, which is primarily supplied by a large market in the city of Talca; it was reported that there is a relationship between chlorpyrifos residue and DAPs in school children. Similar to Thailand, free meals are provided to students in low-income public schools in Chile, and kids generally eat fresh fruit at school [33].

## 5. Conclusions

The correlation between OP residues in vegetables from local wet markets and DAPs in urine samples from school children in four major regions of Thailand is first reported in the present study. The findings demonstrated that OPs residue can be detected in veggies and that children were equally at risk of exposure in four parts of Thailand.

## Figures and Tables

**Table 1:** Demographical data participated school children in rural areas.

Sex	Male	Female	Total	Minimum	Maximum
*n*	231	164	395	-	-
Age, years	9.73 ± 2.74	9.50 ± 2.63	9.601 ± 2.67	5	15
Weight, kg	32.79 ± 12.33	31.18 ± 13.88	31.90 ± 13.18	15	86
Height, cm	134.73 ± 18.05	132.95 ± 15.63	133.74 ± 16.85	100	175
BMI, kg/m^2^	17.45 ± 3.04	16.93 ± 4.33	17.16 ± 3.80	12.4	37.22

**Table 2:** Comparison of residue detection frequency by province based on the vegetable.

Province	Vegetables	N	Detection Frequency (%)							
			Diazinon	Dimethoate	Pirimiphos-Methyl	Chlorpyrifos	Profenofos	Ethion	Triazophos	Total OPs
CM	Cabbage	10	60	0	0	10	0	0	40	70
	Yard long bean	10	0	0	0	90	0	20	60	100
	Chinese cabbage	10	0	90	0	100	0	0	40	100
	Kale	10	70	0	0	90	10	0	20	100
	Carrot	10	10	0	0	100	10	10	0	100
	Total	50	28	18	0	78	4	6	32	94
PT	Cabbage	12	66.7	0	0	8.3	0	0	0	66.7
	Morning glory	12	33.3	33.3	0	91.7	8.3	0	0	100
	Kale	12	83.3	0	0	41.7	41.7	0	8.3	83.3
	Carrot	12	0	0	0	66.7	0	0	0	66.7
	Pak Choi	12	16.7	33.3	0	100	16.7	0	0	100
	Total	60	40	13.3	0	61.7	13.3	0	1.7	83.3
SK	Cabbage	8	100	0	0	37.5	0	0	12.5	100
	Chinese cabbage	9	33.3	77.8	0	100	11.1	0	33.3	100
	Morning glory	8	0	0	0	100	0	0	0	100
	Thai eggpant	8	0	0	12.5	100	0	0	0	100
	Tomato	8	0	0	0	87.5	0	12.5	0	87.5
	Kale	10	90	0	0	90	10	0	30	90
	Total	51	39.2	13.7	2	86.3	3.9	2	13.7	96.1
PNG	Cabbage	12	41.7	0	0	33.3	0	0	0	66.7
	Yard long bean	12	8.3	25	0	91.7	0	0	0	91.7
	Chinese cabbage	12	0	75	0	100	0	0	0	100
	Thai eggpant	12	16.7	0	0	83.3	0	16.7	0	100
	Cucumber	12	0	8.3	8.3	91.7	0	25	0	100
	Total	60	13.3	21.7	1.7	80	0	8.3	0	91.7

Abbreviations: CM: Chiang Mai, PT: Pathum Thani, SK: Sakon Nakhon, PNG: Phang Nga and Total OPs: The sum of 7 types of organophosphate pesticide detection frequency (diazinone, dimethoate, pirimiphos-methyl, chorpyrifos, profenofos, ethion, and triazophos).

**Table 3:** OPs residue detection frequency and concentrations by province.

Province	OPs Residue	Detection		Mean	SD	Median	IQR (mg/kg)	
		(*n*)	%	(mg/kg)		(mg/kg)	1st	3rd
CM	Diazinon	14	28	0.2216	0.2848	0.039	0.0028	0.5332
	Dimethoate	9	18	0.0347	0.0567	0.0114 ^a^	0.0081	0.0355
	Pirimiphos-methyl	0	0	-	-	-	-	-
	Chlorpyrifos	39	78	0.0263	0.0734	0.0031 ^c^	0.0025	0.0061
	Profenofos	2	4	0.0061	0.0011	0.0061	0.0053	-
	Ethion	3	6	0.0103	0.0098	0.0056	0.0037	-
	Triazophos	16	32	0.0038	0.0016	0.0034	0.0027	0.0042
PT	Diazinon	24	40	0.3525	0.4627	0.0912	0.0137	0.6669
	Dimethoate	8	13.3	0.1623	0.1224	0.1637 ^a,b^	0.0609	0.2118
	Pirimiphos-methyl	0	0	-	-	-	-	-
	Chlorpyrifos	37	61.7	0.0202	0.0849	0.0021 ^c,d,e^	0.0018	0.0026
	Profenofos	8	13.3	0.2889	0.5554	0.0502	0.0031	0.4188
	Ethion	0	0	-	-	-	-	-
	Triazophos	1	1.7	0.0021	-	0.0021	0.0021	0.0021
SK	Diazinon	20	39.2	0.2199	0.2625	0.0754	0.012	0.3976
	Dimethoate	7	13.7	0.0354	0.0167	0.0413	0.0159	0.0499
	Pirimiphos-methyl	1	2	0.0021	-	0.0021	0.0021	0.0021
	Chlorpyrifos	44	86.3	0.0057	0.011	0.0025 ^d^	0.0021	0.0047
	Profenofos	2	3.9	0.0399	0.0516	0.0399	0.0034	-
	Ethion	1	2	0.0035	-	0.0035	0.0035	0.0035
	Triazophos	7	13.7	0.0027	0.0007	0.003	0.0022	0.0032
PNG	Diazinon	8	13.3	0.6951	0.7643	0.4316	0.0606	1.2346
	Dimethoate	13	21.7	0.0381	0.0468	0.0193 ^b^	0.0091	0.0502
	Pirimiphos-methyl	1	1.7	0.0024	-	0.0024	0.0024	0.0024
	Chlorpyrifos	48	80	0.0102	0.0322	0.0029 ^e^	0.0022	0.0038
	Profenofos	0	0	-	-	-	-	-
	Ethion	5	8.3	0.0462	0.0702	0.0085	0.0042	0.1071
	Triazophos	0	0	-	-	-	-	-

Abbreviations: Interquartile range (IQR); 1st quartile–3rd quartile, value followed by the same letters (a–e) in the same column are significantly different at <0.05 by Kruskal–Wallis test, post hoc *p*-value, *p*-value adjusted with the Bonferroni method. CM: Chiang Mai, PT: Pathum Thani, SK: Sukhothai, and PNG: Phang Nga.

**Table 4:** DAPs detection frequency and concentration by province.

Province	Biomarkers	Detection		Mean	SD	Median	IQR	
							(µmole/mole Creatinine)	
		*n*	%	(µmole/mole Creatinine)		(µmole/mole Creatinine)	1st	3rd
CM	DMP	13	13.1	7.291	5.09	5.053 a	3.419	9.92
	DMTP	45	45.5	1.498	2.656	0.445	0.256	1.324
	DMDTP	17	17.2	0.407	0.382	0.231 b	0.16	0.516
	DEP	80	80.8	2.413	2.682	1.635 c	0.557	2.987
	DETP	94	94.9	1.48	2.942	0.535	0.313	1.185
	DEDTP	18	18.2	1.141	2.874	0.434	0.242	0.705
	DMs	48	48.5	3.523	6.363	0.786	0.276	3.787
	DEs	97	98	3.636	5.671	1.887	0.744	4.254
	DAPs	97	98	5.379	7.839	2.609 d	0.804	6.187
PT	DMP	16	15.8	24.26	22.944	16.376 a	8.834	31.188
	DMTP	44	43.6	4.451	10.989	0.659	0.381	1.773
	DMDTP	14	13.9	0.658	0.607	0.44	0.218	0.894
	DEP	80	79.2	2.08	2.769	0.853	0.363	2.248
	DETP	96	95	1.523	3.674	0.548	0.35	1.032
	DEDTP	13	12.9	0.628	0.676	0.422	0.25	0.741
	DMs	51	50.5	11.633	21.393	0.922	0.413	14.381
	DEs	99	98	3.24	5.301	1.19	0.725	2.918
	DAPs	99	98	9.233	18.317	1.557	0.992	6.659
SK	DMP	3	3.1	5.924	6.617	2.747	1.495	
	DMTP	31	31.6	1.993	5.346	0.471	0.234	0.714
	DMDTP	7	7.1	1.347	1.124	1.182 b	0.531	1.551
	DEP	72	73.5	1.348	1.821	0.735 c	0.302	1.632
	DETP	93	94.9	0.865	1.226	0.515	0.341	0.991
	DEDTP	19	19.4	0.405	0.173	0.361	0.266	0.453
	DMs	34	34.7	2.617	7.425	0.484	0.252	1.665
	DEs	95	96.9	1.949	2.458	1.158	0.511	2.433
	DAPs	97	99	2.826	6.032	1.596 d	0.663	2.915
PNG	DMP	4	4.1	10.221	5.597	9.322	5.43	15.912
	DMTP	46	47.4	1.347	2.728	0.574	0.392	1.136
	DMDTP	15	15.5	0.536	0.379	0.495	0.239	0.603
	DEP	55	56.7	2.048	2.754	0.838	0.424	3.25
	DETP	90	92.8	1.464	2.958	0.563	0.399	1.058
	DEDTP	8	8.2	0.544	0.448	0.391	0.321	0.498
	DMs	50	51.5	2.218	4.061	0.706	0.418	1.775
	DEs	93	95.9	2.674	4.581	1.044	0.585	2.412
	DAPs	93	95.9	3.867	5.853	1.448	0.902	4.006

Abbreviations: Interquartile range (IQR); 1st quartile–3rd quartile, value followed by the same letters (a–d) in the same column are significantly different at <0.05 by Kruskal–Wallis test, post-hoc *p*-value, *p*-value adjusted with the Bonferroni method. DMP: dimethyl phosphate, DMTP: dimethyl thiophosphate, DMDTP: dimethyl dithiophosphate, DEP: diethyl phosphate, DETP: diethyl thiophosphate, and DEDTP: diethyl dithiophosphate, DMs: DMP + DMTP + DMDPT, DEs: DEP + DETP + DEDTP, DAPs: DMs + DEs, CM: Chiang Mai, PT: Pathum Thani, SK: Sukhothai, and PNG: Phang Nga.

**Table 5:** Organophosphate pesticides and DAP degradation products in urine *.

Organophosphate Pesticide	DAPs Metabolites in Urine
Chlorpyrifos	DEP, DETP
Diazinon	DEP, DETP
Dimethoate	DMP, DMTP, DMDTP
Ethion	DEP, DETP, DEDIP
Pirimiphos-methyl	DMP, DMTP
Triazophose	DEP
Profenophos	Not produce any DAPs

* Quirós-Alcalá, L. et al., 2012 [26].

## Data Availability

Not applicable.
